# Characteristics of changes in plasma proteome profiling after sleeve gastrectomy

**DOI:** 10.3389/fendo.2024.1330139

**Published:** 2024-02-05

**Authors:** Yuying Zhang, Chenye Shi, Haifu Wu, Hongmei Yan, Mingfeng Xia, Heng Jiao, Di Zhou, Wei Wu, Ming Zhong, Wenhui Lou, Xin Gao, Hua Bian, Xinxia Chang

**Affiliations:** ^1^ Department of Endocrinology and Metabolism, Zhongshan Hospital, Fudan University, Shanghai, China; ^2^ Department of General Surgery, Zhongshan Hospital, Fudan University, Shanghai, China; ^3^ Department of Anesthesiology, Zhongshan Hospital, Fudan University, Shanghai, China; ^4^ Department of Critical Care Medicine, Zhongshan Hospital, Fudan University, Shanghai, China

**Keywords:** bariatric surgery, proteomics, morbid obesity, metabolism, weight loss

## Abstract

Bariatric surgery (BS), recognized as the most effective intervention for morbid obesity and associated metabolic comorbidities, encompasses both weight loss-dependent and weight loss-independent mechanisms to exert its metabolic benefits. In this study, we employed plasma proteomics technology, a recently developed mass spectrometric approach, to quantitatively assess 632 circulating proteins in a longitudinal cohort of 9 individuals who underwent sleeve gastrectomy (SG). Through time series clustering and Gene Ontology (GO) enrichment analysis, we observed that complement activation, proteolysis, and negative regulation of triglyceride catabolic process were the primary biological processes enriched in down-regulated proteins. Conversely, up-regulated differentially expressed proteins (DEPs) were significantly associated with negative regulation of peptidase activity, fibrinolysis, keratinocyte migration, and acute-phase response. Notably, we identified seven proteins (ApoD, BCHE, CNDP1, AFM, ITIH3, SERPINF1, FCN3) that demonstrated significant alterations at 1-, 3-, and 6-month intervals post SG, compared to baseline. These proteins play essential roles in metabolism, immune and inflammatory responses, as well as oxidative stress. Consequently, they hold promising potential as therapeutic targets for combating obesity and its associated comorbidities.

## 1 Introduction

With drastic shifts in the Chinese social economy and mode of life, the prevalence of obesity in China has escalated from 3.1% in 2004 to 8.1% in 2018 (1). Obesity, as a chronic metabolic disease, is closely associated with various comorbidities, including but not limited to type 2 diabetes mellitus (T2DM), hypertension, hyperlipidemia, metabolic-associated fatty liver disease (MAFLD), cardiovascular diseases, several cancers, and obstructive sleep apnea. These conditions pose a substantial burden on public health and place increasing strain on the healthcare system (2–5). The management of obesity entails lifestyle modifications, drug therapy, and bariatric surgery. However, the former two interventions yield limited effects on sustainable weight loss and are accompanied by high rates of recidivism. In contrast, bariatric surgery has demonstrated its effectiveness as the most appropriate intervention for achieving long-term weight loss and improving obesity-related comorbidities (6). Recent guidelines recommend bariatric surgery for patients who have not achieved success through lifestyle intervention and possess a BMI exceeding 35 kg/m^2^ or a BMI of at least 30 kg/m^2^ with an obesity-related comorbidity (7, 8). Initially, it was assumed that weight loss was attributed solely to reduced energy intake and impaired absorption. However, the discovery of immediate weight-independent outcomes following surgery has shed light on underlying mechanisms. Enhanced central appetite control, gut peptide release, alterations in microbiota, bile acids, insulin sensitivity, and insulin secretion have been recognized as contributors to improved metabolic health (9–12). Nonetheless, the weight loss-dependent mechanisms underlying the various metabolic improvements observed in individuals who have undergone BS have yet to be elucidated.

In order to provide insights on the potential mechanism of metabolic improvement after BS, which may make contributions to developing safer nonsurgical interventions in obesity or other metabolic diseases, we identified the changes in the plasma proteome after SG.

## 2 Materials and methods

### 2.1 Patients

A total of nine individuals (4 males and 5 females) underwent SG at Zhongshan Hospital, Fudan University, between June 2014 and June 2017. The selection of patients for bariatric surgery followed the guidelines set by the Chinese Society for Metabolic and Bariatric Surgery (CSMBS). These guidelines stipulated that patients must meet the following criteria: (1) age between 18 and 60 years, and (2) a BMI ≥ 32 kg/m2 or a BMI ≥ 27.5 kg/m2 with one or more comorbidities such as type 2 diabetes, dyslipidemia, or hypertension. Nonsurgical treatments had been unsuccessful in achieving the desired weight loss or managing complications. All surgeries were performed by the same surgeon, HF Wu. Follow-up assessments were conducted at baseline (pre-surgery), 1-month post-surgery, 3-month post-surgery, and 6-month post-surgery. During each follow-up visit, anthropometric measurements, clinical biochemical parameters, and blood serum proteomes were collected.

### 2.2 Anthropometric and serum biochemical measurements

Height and weight were measured without shoes or outer clothing. Venous blood samples for biochemical measurements were collected after fasting for at least 12 hours. Serum total cholesterol (TC), high-density lipoprotein (HDL) cholesterol, and triglyceride (TG) levels were measured by an oxidase method on a model 7600 automated bioanalyzer (Hitachi, Tokyo,Japan). Low-density lipoprotein (LDL) cholesterol was calculated using the Friedewald equation. Glycosylated hemoglobin (HbA1c) levels were determined using high-pressure liquid chromatography on the Variant™ II machine (Bio-Rad, Hercules, CA, USA). Plasma glucose was measured by a glucose oxidase method. Serum insulin concentrations were determined by Auto DELFIA fluoroimmunoassay. Homeostatic model assessment of insulin resistance (HOMA-IR) was calculated as fasting plasma glucose (mmol/L) × fasting insulin (mU/mL)/22.5. Liver fat content defined as the ratio of area lipid (AL) and AL plus area water (AW) was measured by proton magnetic resonance spectroscopy (^1^H-MRS) (13).

### 2.3 Serum protein extraction and trypsin digestion

Serum samples were first depleted the top 14 abundant proteins using an immunodepleting kit (Thermo Fisher) according to the manufacturer’s instructions. The depleted serum was digested by trypsin at an enzyme to protein mass ratio of 1:25 overnight at 37°C prior to extraction and desiccation.

### 2.4 LC-MS/MS analysis

Samples were measured using LC-MS instrumentation consisting of an EASY-nLC 1200 ultra-high-pressure system (Thermo Fisher Scientific) coupled to a Fusion Lumos Orbitrap (Thermo Fisher Scientific) via a nano-electrospray ion source (Thermo Fisher Scientific). The peptides were dissolved with 12 μl loading buffer (0.1% formic acid in water), and 5 μl was loaded onto a 100 um I.D. × 2.5 cm, C18 trap column at a maximum pressure 280 bar with 14 μl solvent A (0.1% formic acid in water). Peptides were separated on 150 um I.D. × 15 cm column (C18, 1.9lm, 120 #A, Dr. Maisch GmbH) with a linear 15-30% Mobile Phase B (ACN and 0.1% formic acid) at 600 nl/min for 75 min. The MS analysis was performed in a data-independent manner (DIA), which consisted of MS1 scan from 300-1,400 m/z at 60k resolution (AGC target 4e5 or 50ms). Then, 30 DIA segments were acquired at 15k resolution with an AGC target 5e4 or 22ms for maximal injection time. The setting “inject ions for all available parallelizable time” was enabled. HCD fragmentation was set to normalized collision energy of 30%. The spectra were recorded in profile mode. The default charge state for the MS2 was set to 3.

### 2.5 Peptide identification and protein quantification

The DIA data were search against UniProt human protein database (updated on 2019.12.17, 20406 entries) using FragPipe (v12.1) with MSFragger (2.2). The mass tolerances were 20 ppm for precursor and 50 mmu for product ions. Up to two missed cleavages were allowed. The search engine set cysteine carbamidomethylation as a fixed modification and N-acetylation and oxidation of methionine as variable modifications. Precursor ion score charges were limited to +2, +3, and +4. The data were also searched against a decoy database so that protein identifications were accepted at a false discovery rate (FDR) of 1%. The results of DIA data were combined into spectra libraries using SpectraST software.

DIA data was analyzed using DIA-NN (v1.7.0). The default settings were used for DIA-NN (Precursor FDR: 5%, Log lev: 1, Mass accuracy: 20 ppm, MS1 accuracy: 10 ppm, Scan window: 30, Implicit protein group: genes, Quantification strategy: robust LC (high accuracy)). Quantification of identified peptides was calculated as the average of chromatographic fragment ion peak areas across all reference spectra libraries. Label-free protein quantifications were calculated using a label-free, intensity-based absolute quantification (iBAQ) approach. We calculated the peak area values as parts of corresponding proteins. The fraction of total (FOT) was used to represent the normalized abundance of a particular protein across samples. FOT was defined as a protein’s iBAQ divided by the total iBAQ of all identified proteins within a sample. The FOT values were multiplied by 10^5^ for the ease of presentation and missing values were imputed with 10^-5^.

### 2.6 Ethics approval and informed consent

The study was approved by the ethics committee of Zhongshan Hospital, Fudan University and adhered to the guidelines of the Declaration of Helsinki. Informed consent was obtained from all participants.

### 2.7 Statistical analysis

Statistical analysis of anthropometric and biochemical measurements was conducted using SPSS (Version 26.0). Prior to analysis, normality of the data was assessed. Normally distributed variables were presented as mean ± standard deviation and paired t-tests were employed to compare continuous variables between groups. Differential analysis of proteomics was performed using paired t-test by R language. Proteins that exhibited differences in expression, indicated by p-values < 0.05 and fold change (FC) values ≥ 1.2 or ≤ 0.83, were defined as differentially expressed proteins (DEPs). Time-series analysis and cluster heatmaps were generated using the Bioinformatics website (https://www.bioinformatics.com.cn/). Pathway enrichment analysis was performed using the DAVID tool (https://david.ncifcrf.gov/). Boxplots depicting the levels of DEPs at four time points were generated using Graphpad Prism (Version 8.0.2). Pearson correlation analysis and partial correlation analysis were conducted using SPSS to examine the relationship between DEPs and specific metabolic components. A significance level of P < 0.05 was employed for all analyses.

## 3 Results

### 3.1 Clinical outcomes

Our study consists of 9 morbidly obese patients with BMI 41.07 ± 5.96kg/m^2^. Anthropometric and serum biochemical measurements of patients were collected at baseline, 1-, 3- and 6-months after SG as displayed in **Table 1**. Obviously, SG has a significant effect on weight loss, as a consistent and statistically significant reduction in BMI was observed at three longitudinal postoperative time points(P<0.001). FBG declined at 1-, 3- and 6-months post-SG but did not reach significance at 3 months compared to baseline. SG resulted in significant decrease in HbA1c at both 3- and 6-months compared to baseline. HOMA-IR, which reflects insulin resistance, declined significantly at all the three time points after surgery. SG resulted in a significant reduction in TG after 1- and 6-months (p < 0.05) but not 3-month. HDL increased significantly at 6-months(P<0.05). ^1^H-MRS is a non-invasive indicator used to assess the degree of hepatic steatosis, and liver fat content measured by ^1^H-MRS declined significantly at 1-, 3- and 6-months(P<0.05).

**Table 1:** Anthropometric and clinical measurements taken from patients before and after bariatric surgery.

	Baseline(mean± SD)	1 months Post-SG(mean± SD)	3 months Post-SG(mean ± SD)	6 months Post-SG(mean ± SD)
Number of samples	9	9	9	9
Sex	4 Males, 5 Females			
Age	37.67 ± 3.83			
BMI(kg/m^2^)	41.07 ± 5.96	35.59 ± 5.41***	32.02 ± 5.07***†††	29.05 ± 5.28***†††‡‡‡
FBG(mmol/L)	7.40 ± 3.60	4.04 ± 0.70*	4.67 ± 1.15	4.21 ± 0.13*
HbA1c(%)	6.48 ± 1.75	5.87 ± 1.16	5.32 ± 0.58*	5.27 ± 0.38*
HOMA-IR	5.64 ± 2.47	1.33 ± 0.56**	1.73 ± 0.77*	1.23 ± 0.70**
TC(mmol/L)	3.86 ± 1.11	3.73 ± 0.83	3.96 ± 0.61	4.23 ± 0.56
TG(mmol/L)	1.95 ± 1.26	1.04 ± 0.28*	1.12 ± 0.32	0.91 ± 0.31*
LDL-c(mmol/L)	2.20 ± 0.46	2.28 ± 0.76	2.34 ± 0.46	2.46 ± 0.51
HDL-c(mmol/L)	1.11 ± 0.16	0.98 ± 0.16	1.10 ± 0.20†	1.35 ± 0.24*†††‡‡‡
SBP(mmHg)	125 ± 14.46	136.33 ± 17.20	120.44 ± 15.92††	125.44 ± 19.77
DBP(mmHg)	82.67 ± 12.03	82.11 ± 12.33	74.89 ± 11.66	78.11 ± 8.21
Liver fat content(%)	38% ± 20%	20% ± 8%*	14% ± 5%*†	8% ± 4%*

SBP, systolic blood pressure; DBP, diastolic blood pressure

* compared with baseline; † compared with 1 month post-SG; ‡ compared with 3 months post-SG.

* † ‡ p<0.05, ** † †‡‡ p<0.01, ***††† ‡ ‡ ‡ p<0.001.

### 3.2 Identification of DEPs and GO enrichment analysis of DEPs

**Figure 1:**
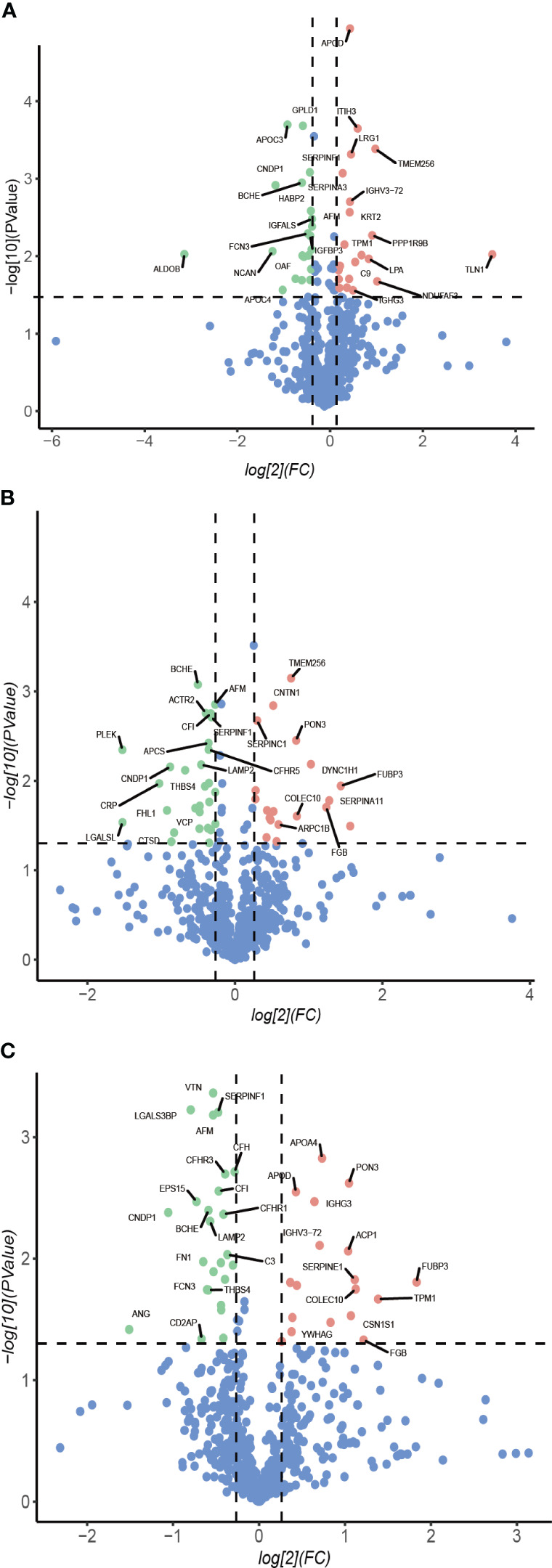
Volcano plot. Protein abundance changes 1-month **(A)**, 3-month **(B)**, 6-month **(C)** post-surgery compared to baseline. Red points are significant and have a FC >1.2, green points are significant and have a FC <-0.83, blue points are not significant.

**Figure 2:**
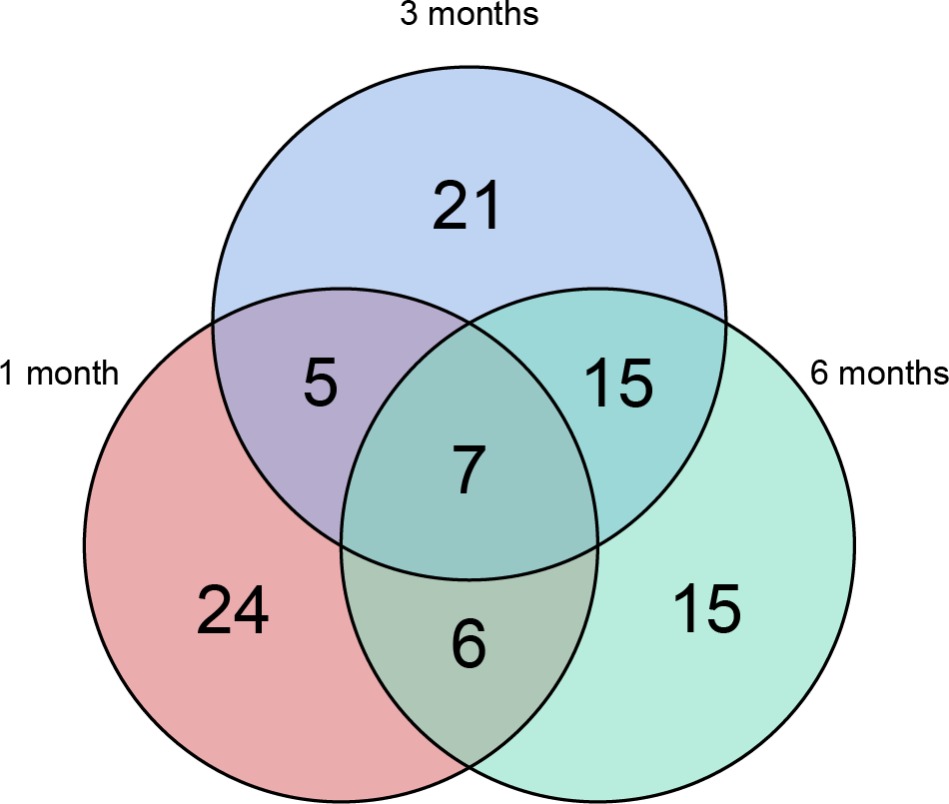
Venn plot. 93 proteins changed significantly throughout the entire postoperative follow-up. 42 proteins showed statistically significant changes at 1-month after surgery comparing with baseline, 48 proteins after 3 months and 43 proteins after 6 months. Relative to pre-surgery samples, 12 proteins were significantly altered at both 1- and 3-months post-surgery, 13 proteins at both 1- and 6-months, 22 proteins at both 3- and 6-months and 7 proteins at all the three time points.

**Figure 3:**
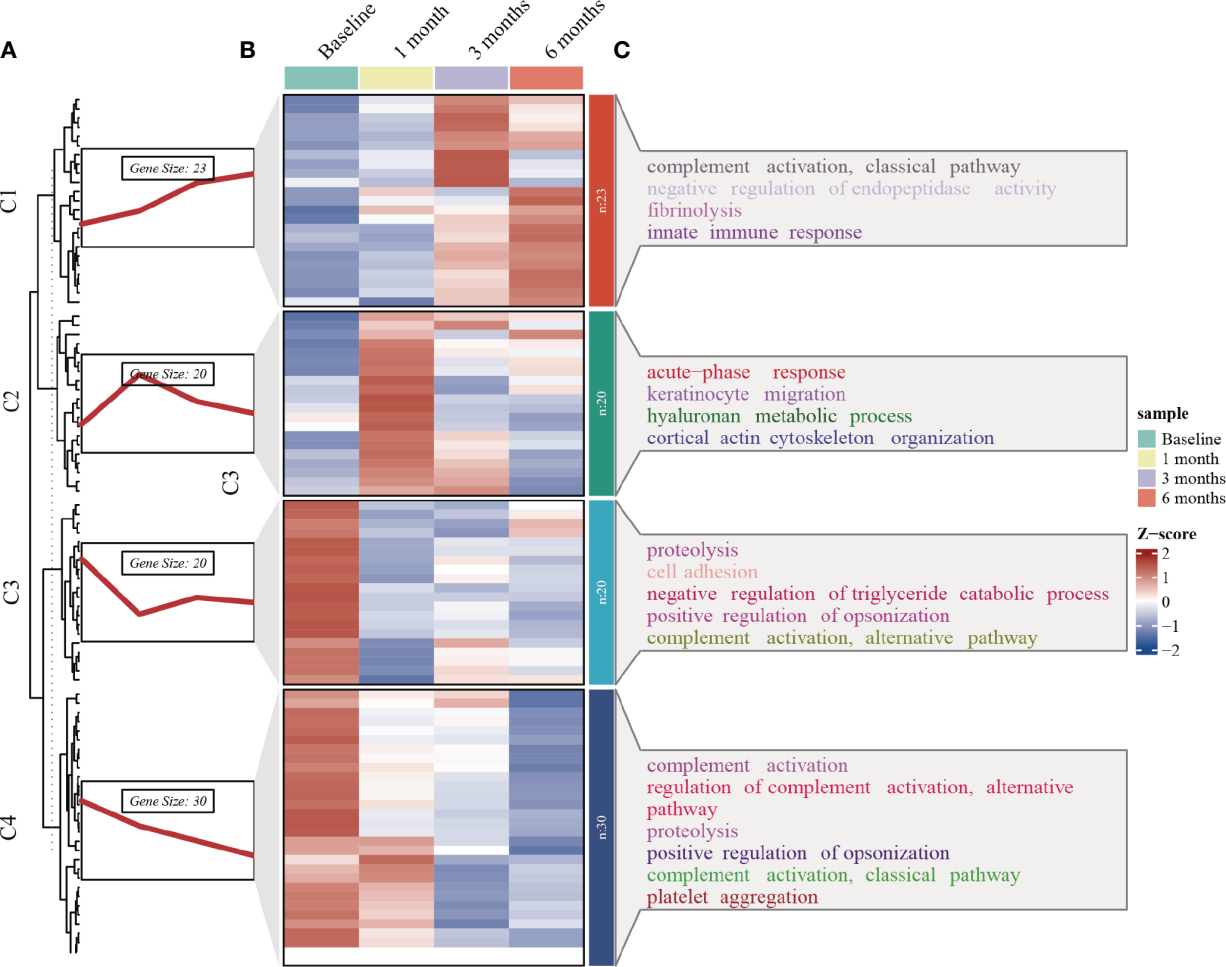
Time series clustering, heat map and GO enrichment analysis performed on 93 DEPs. **(A)** Time series clustering. Based on the core algorithm, Fuzzy C-means Clustering, the proteins with similar expression patterns were classified into 4 clusters. **(B)** Hierarchical clustering of the plasma proteins in the four clusters. **(C)** BP analysis in GO annotation for each of the protein clusters and top terms enriched with the lowest p-value are labeled.

Longitudinal plasma proteomics is of great value in analysis. LC-MS proteomics was conducted on 36 plasma samples from 9 patients at four time points: baseline, 1-month post-surgery, 3 months post-surgery, and 6 months post-surgery. A cumulative total of 632 proteins were identified in these plasma proteomes. Through differential expression analysis, the levels of 93 proteins were found to significantly change throughout the entire postoperative follow-up. Specifically, 42 proteins exhibited statistically significant changes at 1-month post-surgery compared to baseline, 48 proteins at 3 months, and 43 proteins at 6 months (**Figure 1**). When compared to pre-surgery samples, 12 proteins showed significant alterations at both 1- and 3-months post-surgery, 13 proteins at both 1- and 6-months, 22 proteins at both 3- and 6-months, and 7 proteins at all three time points (**Figure 2**). Conducting time-series cluster analysis on the all 93 DEPs, these proteins were classified into four main groups based on their response to the surgery. Cluster 1, consisting of 23 proteins, displayed a sustained increase throughout the follow-up period, while Cluster 4 (30 proteins) exhibited a consistent decline. Cluster 2 (20 proteins) initially increased at the first post-operative time point before returning to baseline levels for the entire 6-month period. The expression of Cluster 3 (20 proteins) decreased at 1-month post-surgery and maintained a lower level than baseline thereafter (**Figure 3A**). The expression patterns of each cluster are visually represented using heat maps (**Figure 3B**). To explore the key biological functions, GO enrichment analysis was conducted separately on the four protein clusters, and the top enriched biological process (BP) terms are presented in **Figure 3C** (**Supplementary Table 1**). The most significantly enriched GO terms in Cluster 1 are complement activation, negative regulation of endopeptidase activity, and fibrinolysis. Cluster 2 is primarily enriched in acute-phase response, hyaluronan metabolic process, and keratinocyte migration. Cluster 3 is mainly involved in proteolysis, cell adhesion, and negative regulation of triglyceride catabolic process. Complement activation, proteolysis, and platelet aggregation are the main GO terms enriched in Cluster 4.

### 3.3 The expression profiles of proteins differentially expressed at all three time points post- surgery

**Figure 4:**
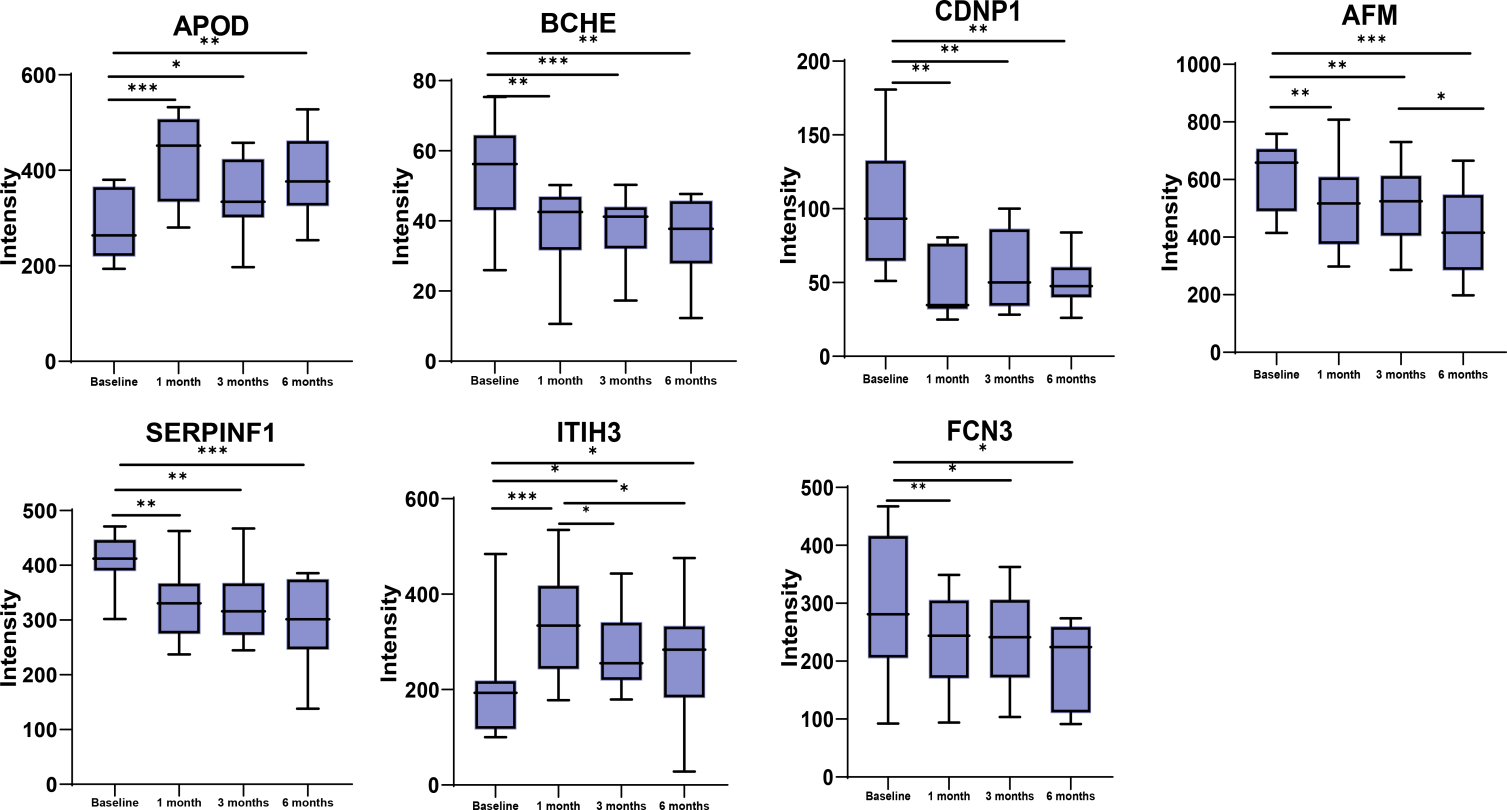
Changes in protein expression between baseline, 1 month, 3 months and 6 months for the 7 DEPs at all the three time points post-surgery. *p<0.05, **p<0.01, ***p<0.001.

Seven proteins were found to be significantly altered at all three postoperative time points compared to the baseline (**Figure 4**). Butyrylcholinesterase (BCHE), carnosine dipeptidase 1(CNDP1), Afamin (AFM), serpin family F member 1 (SERPINF1), and ficolin 3(FCN3) exhibited a significant decrease at 1-, 3-, and 6-months compared to the baseline. On the other hand, Apolipoprotein D (ApoD) and inter-alpha-trypsin inhibitor heavy chain 3(ITIH3) showed an increase at 1-month, followed by a decline but still above the baseline levels at 3- and 6-months. To investigate the potential role of these DEPs in metabolism, correlation analysis was performed between the 7 DEPs and BMI, HOMA-IR, and liver fat content. Moreover, age, sex, and BMI were accounted for as confounding factors that could directly influence HOMA-IR and liver fat content (**Table 2**). In our study, SERPINF1 demonstrated a significant positive correlation with BMI. CNDP1, AFM and SERPINF1 showed a significant association with HOMA-IR. BCHE, CNDP1, AFM, SERPINF1, ITIH3, and FCN3 exhibited either positive or negative correlations with liver fat content. After adjusting for age, gender, and BMI, the correlation between AFM, ApoD, and liver fat content did not reach statistical significance. Furthermore, the correlations between the other 86 significantly changed proteins during the entire postoperative follow-up and clinical parameters, such as BMI, HOMA-IR, and hepatic fat content, are presented in **Supplementary Table 2**.

**Table 2:** Correlation of serum levels of 7 proteins differentially expressed at all three time points post-surgery compared to baseline to BMI, HOMA-IR and liver fat content.

	BMI	HOMA-IR	liver fat content	HOMA-IR(adjusted by age, sex and BMI)	liver fat content (adjusted by age, sex and BMI)
APOD	-0.136	-0.33	-0.293	-0.339	-0.225
BCHE	0.016	0.233	0.668^**^	0.133	0.485^*^
CNDP1	0.281	0.468^**^	0.606^**^	0.441^*^	0.532^**^
AFM	-0.115	0.399^*^	0.539^**^	0.482^*^	0.313
SERPINF1	0.601^***^	0.509^**^	0.436^*^	0.24	0.29
ITIH3	0.294	-0.315	-0.608^**^	-0.474^*^	-0.652^**^
FCN3	-0.04	0.108	0.596^**^	0.072	0.451^*^

* p<0.05, ** p<0.01, ***p<0.001. Partial correlation analysis between serum levels of 7 DEPs and HOMA-IR and liver fat content adjusted for age, sex and BMI.

## 4 Discussion

Bariatric surgery elicits profound metabolic changes, yet the underlying molecular mechanisms remain elusive. In this study, we performed plasma proteome profiling before and after SG. Our objective was to gain insights into the potential mechanisms driving metabolic improvements following SG and identify protein markers with potential functional relevance, which could aid in the development of safer non-surgical interventions for obesity and other metabolic disorders. We observed significant alterations in the levels of several metabolism-related proteins in the bloodstream at 1-, 3-, and 6-months post-surgery, with seven proteins altered at all three time points. Through time-series analysis and GO enrichment analysis of DEPs, we identified complement activation, proteolysis, and negative regulation of triglyceride catabolic process as the most significantly enriched pathways among down-regulated proteins. On the other hand, negative regulation of peptidase activity, fibrinolysis, keratinocyte migration, and acute-phase response were the main significant pathways enriched among up-regulated DEPs. The proteins ApoD, BCHE, CNDP1, AFM, SERPINF1, ITIH3, and FCN3 were consistently differentially expressed across all post-surgery periods compared to baseline.

Throughout the 6-month follow-up period, all 9 participants experienced sustained weight loss. As anticipated, levels of HbA1c and HOMA-IR, indicators of blood glucose control and insulin resistance respectively, decreased at both the 3- and 6-month marks after surgery, with the exception of a non-statistically significant decrease in HOMA-IR at 3 months. This suggests that the remission of diabetes following SG may be partly attributed to a reduction in insulin resistance. Notably, improvements in blood lipid profiles were observed during postoperative follow-up, including a decrease in triglycerides and an increase in HDL levels. Recently ^1^H-MRS has emerged as an invaluable noninvasive tool for identifying patients with hepatic steatosis and assessing changes in liver fat content. The decrease in liver fat content at 1-, 3-, and 6-month intervals post-SG indicates the remission of hepatic steatosis, which can be attributed not only to weight loss but also to potential physiological mechanisms. Overall, the improvements in human metabolism following SG were significant.

The 93 DEPs were categorized into 4 clusters based on changes in expression levels using time series analysis. Further functional enrichment analyses were conducted to investigate the role of these DEPs (**Figure 3**). GO annotation analysis revealed that complement activation, a crucial component of the innate immune system, was the most significant pathway enriched in the consistently downregulated DEPs (cluster 4) following SG. While the complement system was initially recognized as the primary defense against microbial invaders, its activation has been associated with increased adipose tissue inflammation, insulin resistance, systemic low-grade inflammation, and endothelial cell dysfunction (14–16). Over time, the involvement of complement proteins in metabolic syndromes such as obesity, diabetes, and atherosclerosis has gradually been unveiled. Complement 3 (C3) serum levels are considered a pro-inflammatory biomarker for insulin resistance in obesity (17), and in our study, C3 serum levels exhibited a decreasing trend overall. However, complement activation was also enriched in the upregulated DEPs (cluster 1). This enrichment was driven by four proteins: Immunoglobulin heavy constant gamma 2 (IGHG2), Immunoglobulin heavy constant gamma 3 (IGHG3), Mannose binding lectin 2 (MBL2), and Complement 9 (C9). No relationship has been established between the first two proteins and metabolic syndrome. Increased serum MBL levels have been shown to activate NF-κB and renal inflammation in the progression of diabetic nephropathy (18). A Danish cohort study of 7,305 individuals found that serum MBL exhibited a U-shaped association with the risk of cardiovascular events in individuals with T2DM, with both low and high MBL expression genotypes correlating with an increased risk of cardiovascular events (19). A study demonstrated significant upregulation of C9 in obese patients compared to non-obese patients, which contradicts our findings (20). Therefore, the significance of complement pathway enrichment in the upregulated proteins is difficult to explain. However, it is likely that changes in complement-related pathways contributed to the improvement of metabolic syndrome following SG. Obesity is characterized by coagulation and hemostasis disorders and is associated with a prothrombotic tendency. The enrichment of increased fibrinolysis (cluster 1) and decreased platelet aggregation (cluster 4) suggests that SG shifts the hemostatic balance towards an antithrombotic direction, thereby reducing the risk of atherothrombosis in obese individuals. As expected, acute phase response and keratinocyte migration, related to wound healing, were enriched in cluster 2, showing an upregulation tendency in the first month following SG before returning to baseline. According to the enriched GO terms of cluster 3, negative regulation of triglyceride catabolic process was downregulated, indicating an increase in lipolysis and consistent with a decrease in serum triglyceride levels after surgery.

The panel of seven proteins identified to change at all three postoperative time points compared to baseline play important roles in metabolism. We have highlighted the functions of each protein in the context of obesity, BS, T2DM and other metabolic disorders below.

ApoD is an atypical apolipoprotein with widespread distribution in various tissues. In the bloodstream, ApoD is predominantly found in high-density lipoproteins (HDL). It serves as a carrier for multiple lipids, including arachidonic acids, cholesterol, and steroids. The biological functions of ApoD are known to be associated with neuroprotection, metabolic processes, and cancer, partly owing to its anti-oxidative stress and anti-inflammatory properties. The protective effect of ApoD has been clearly demonstrated at both cellular and organism levels (21–23). ApoD safeguards against increased lipid peroxidation resulting from oxidative stress (24). Overexpression of human ApoD resulted in a reduction of plasma IL-6 and TNF-α levels in two mouse models. Further investigation revealed that ApoD regulates lipid mediators and osteopontin in an anti-inflammatory manner (25). The role of ApoD in the regulation of glucose and lipid metabolism is not yet fully understood. In a recent study, higher APOD levels were found to be linked to better metabolic health and inflammatory state in the round ligament of morbidly obese women (26). Conversely, another study showed that mice overexpressing human ApoD developed hepatic steatosis and insulin resistance over time, with the accumulation of arachidonic acid and overactivation of PPARγ believed to contribute to the pathogenesis (21). In our study, postoperative ApoD expression showed an increasing trend, which may be associated with the beneficial effects of bariatric surgery.

BCHE is widely distributed in both central and peripheral tissues, including the brain, liver, serum, and skin. Since its neuroprotective effects were first proven in 1991 (27), BCHE has garnered increasing interest. Numerous studies have demonstrated that genetic variations in BCHE influence its activity and are associated with BMI and metabolic syndrome. In obese individuals, BCHE activity is positively correlated with body weight and the components of metabolic syndrome (28–31). Knockout mice for BCHE, when subjected to a high-fat diet, exhibited greater weight gain, hepatic fat accumulation, and higher levels of inflammatory markers (32). This may be attributed to the fact that BCHE, being a serine hydrolase, hydrolyzes ghrelin and subsequently reduces its plasma levels, thereby suppressing ghrelin signaling (33). In our study, we observed a downward trend in BCHE expression following bariatric surgery. We speculate that this change in expression may be more likely a consequence rather than a cause of the benefits associated with sleeve gastrectomy. Overall, BCHE holds promise as a potential target for treating obesity, but further research is warranted.

Multiple studies conducted on various ethnic groups have demonstrated the association between the CNDP1 5 leucine/5 leucine (5-5) polymorphism and the risk of developing diabetic nephropathy (DN) (34–37). The CNDP1 gene encodes carnosinase 1 (CN1), which primarily hydrolyzes carnosine, a dipeptide containing histidine, and its other metabolic functions are largely unknown (38). Carnosine serves various beneficial roles, such as anti-inflammation, resistance to oxidation, inhibition of glycation, and clearance of active carbonyl groups that contribute to organ damage in diabetes. Animal studies have demonstrated the protective effects of carnosine in nephropathy, particularly in chronic kidney disease, ischemia/reperfusion-induced acute renal failure, DN, and drug-induced nephrotoxicity (39). Research has also shown the remission of DN following bariatric surgery, leading to improved renal function and reduced albuminuria (40–43). In our observations, we noted a decrease in CNDP1 levels after SG, which may contribute to the remission of DN. However, the specific relationship between CNDP1 polymorphism, CN1 activity, and carnosine levels remains unclear. Further research is necessary to establish CNDP1 and carnosine as potential therapeutic targets for DN.

AFM, a member of the albumin gene family, functions as a carrier of vitamin E. Circulating afamin is primarily synthesized in the liver, but high concentrations have also been detected in cerebrospinal, ovarian follicular, and seminal fluids. Numerous studies have established a strong association between AFM and metabolic syndrome, along with its related disorders. Transgenic mice that overexpressed the human afamin gene exhibited weight gain (44). A large population-based cohort study found a robust link between afamin and insulin resistance, as well as the prevalence and incidence of type 2 diabetes (45). In patients with MAFLD, serum afamin concentrations were elevated and independently predicted the development of MAFLD (46, 47). AFM has also been associated with gestational diabetes, diabetic nephropathy, and various types of cancer (48). A recent study observed a non-significant reduction in AFM levels among patients who underwent weight loss after BS (49). In our study, we observed a sustained and significant decline in AFM levels. Importantly, serum AFM concentration exhibited a positive correlation with HOMA-IR and liver fat content, although the correlation with liver fat content did not reach significance after adjusting for BMI, age, and sex. However, the pathophysiology underlying the involvement of AFM in metabolic syndrome remains poorly understood. AFM shows promise as a potential target for the treatment of metabolic syndrome, but further research is warranted.

SERPINF1, also called pigment epithelium-derived factor (PEDF), is a member of the serine proteinase inhibitor family. As an adipocyte secretory factor, PEDF has been illustrated to have neuroprotective, anti-fibrotic and anti-inflammatory properties, and it is a potent endogenous angiogenic inhibitor (50). PEDF was originally isolated from cultured human fetal retinal pigment epithelial cells and has been certified as a protecter of retinal neurons and a target to suppress choroidal neovascularization (51). PEDF also play an important role in metabolic disorders. Circulating levels of PEDF are elevated in various metabolic disorders, such as obesity and diabetes, and declined upon weight loss and insulin sensitization. Controversially, PEDF is demonstrated to induce insulin resistance in obesity in partial researches, and lipotropic ectopic deposition, impaired insulin signal transduction, mitochondrial dysfunction and inflammation are proposed to be potential mechanisms (52–54). While according to some other restudies, PEDF reduces and reverses HFD-induced obesity, hepatic steatosis and hepatic fibrosis and improves insulin sensitivity and white adipose tissue inflammation *in vivo (* 55, 56).In our cohort, we observed a significant association between PEDF levels and three time points. Specifically, PEDF circulating levels were positively correlated with BMI, HOMA-IR, and liver fat content. However, after adjusting for age, sex, and BMI, the association with HOMA-IR and liver fat content did not reach statistical significance. These findings highlight PEDF as a promising target for the treatment of metabolic syndrome, although further studies are needed to elucidate its precise mechanisms and functions.

ITIH3 encodes the heavy chain subunit of the pro-α-trypsin inhibitor complex, which plays a role in promoting the stability of the extracellular matrix by covalently bonding with hyaluronic acid (57). Polymorphisms of ITIH3 have been associated with an increased risk for schizophrenia and major depressive disorder (58, 59). ITIH3 has also demonstrated antitumoral and antimetastatic properties, making it a potential biomarker for various types of cancer such as pancreatic cancer, prostate cancer, stomach cancer, and lung adenocarcinoma (60–62). Elevated expression levels of ITIH3 in the bloodstream have been found in cases of gestational hypertension and preeclampsia, suggesting its role as a mediator of thrombo-inflammation in these conditions (63). A study conducted on rats revealed that ITIH3 in the bloodstream of obesity-prone males and females fed a high-fat diet exhibited opposite regulatory patterns, with up-regulation in males and down-regulation in females, in comparison to rats fed a normal diet or obesity-resistant rats on a high-fat diet (64). In our cohort, we observed that ITIH3 was up-regulated at 1-month after SG and subsequently down-regulated, irrespective of gender. However, there is a scarcity of data regarding the role of ITIH3 in obesity and metabolic disorders. Some researchers have suggested the potential of targeting ITIH3 as a therapeutic approach for obesity.

Ficolins, a group of pattern recognition molecules that include FCN1, FCN2, and FCN3, have the ability to bind to N-acetylglucosamine, N-acetylgalactosamine, and N-acetyl-neuraminic acid residues on the surface of microbes, subsequently activating the complement lectin pathway. Growing evidence suggests a relationship between the complement system and the development of diabetic nephropathy. The potential mechanism involves hyperglycemia-induced activation of the lectin pathway and dysfunction of complement regulatory proteins, ultimately leading to excessive activation of the complement pathway. This, in turn, facilitates spontaneous complement attack (65). An 18-year follow-up study discovered that elevated levels of FCN3 in circulation were associated with a higher incidence of micro- or macroalbuminuria in patients newly diagnosed with type 1 diabetes (66). Furthermore, increased levels of ficolin-3 were observed in the vitreous humor and serum of patients with proliferative diabetic retinopathy (67). A recent study indicated that elevated FCN3 levels were linked to an increased risk of diabetes-related mortality (68). However, some studies suggest that low serum levels of FCN3 are associated with insulin resistance and diabetic peripheral neuropathy (69, 70). Our research identified a significant decrease in FCN3 levels, which may have a beneficial impact on reducing long-term complications associated with diabetes. However, no correlation was found between FCN3 and insulin resistance. Exploring novel approaches to modulate the complement system could potentially offer opportunities for preventing or slowing the progression of diabetic complications.

In our study, GO enrichment analysis of protein clusters classified by expression patterns over time was performed to find out the key biological processes, and we have identified significant differences of a panel of metabolically relevant proteins in bloodstream after SG by longitudinal follow-up at 4 time points, which is a highlight of our research. Admittedly, the main limitation is the sample size, we collected data only on 9 individuals. Beyond that, we only described changes in proteome profiling after SG in plasma rather than in hepatic tissues, adipose tissues. Considerably more work will need to be done to explore the set of proteins, which has been on the agenda.

## 5 Conclusion

In a nutshell, plasma proteome analysis is a powerful technique for studying the effects of physiological interventions, such as SG. Using longitudinal sampling, we identified seven proteins that were significantly changed at 1-, 3-, and 6-months after SG compared with baseline. The panel of proteins are closely related to metabolic, immune and inflammatory responses and oxidative stress. We discussed the potential link of these proteins with benefits from SG. These proteins may become therapeutic targets for the treatment of metabolic syndrome, and of course further research support is needed.

## Data availability statement

The datasets presented in this study can be found in online repositories. The names of the repository/repositories and accession number(s) can be found in the article/**Supplementary Material**.

## Ethics statement

The studies involving humans were approved by the ethics committee of Zhongshan Hospital, Fudan University. The studies were conducted in accordance with the local legislation and institutional requirements. The participants provided their written informed consent to participate in this study. Written informed consent was obtained from the individual(s) for the publication of any potentially identifiable images or data included in this article.

## Author contributions

YZ: Formal analysis, Visualization, Writing – original draft. CS: Formal analysis, Writing – original draft. HW: Writing – review & editing. HY: Data curation, Funding acquisition, Writing – original draft. MX: Data curation, Software, Writing – original draft. HJ: Data curation, Writing – original draft. DZ: Data curation, Writing – original draft. WW: Resources, Writing – original draft. MZ: Resources, Writing – original draft. WL: Resources, Writing – original draft. XG: Investigation, Methodology, Project administration, Supervision, Writing – review & editing. HB: Investigation, Methodology, Project administration, Supervision, Writing – review & editing. XC: Investigation, Methodology, Project administration, Supervision, Writing – review & editing.
