# Supplementary material for: Characteristics of changes in plasma proteome profiling after sleeve gastrectomy
Source: Front Endocrinol (Lausanne). 2024 Feb 5;15:1330139. doi: 10.3389/fendo.2024.1330139 (PMC10875463; doi:10.3389/fendo.2024.1330139)
Supplement: Supplementary file 1 [file Table_1.docx]

Supplementary Material

Characteristics of changes in plasma proteome profiling after sleeve gastrectomy

**Yuying Zhang^1†^, Chenye Shi^2†^, Haifu Wu^2†^, Hongmei Yan^1^, Mingfeng Xia^1^, Heng Jiao^2^, di Zhou^3^, Wei Wu^4^, Ming Zhong^4^, Wenhui Lou^2^, Xin Gao^1*^, Hua Bian^1*^, Xinxia Chang^1*^**

*** Correspondence:**

Xin Gao

Department of Endocrinology, Zhongshan Hospital, Fudan University, NO.180 Fenglin Road, Shanghai 200032, China.

Email: zhongshan_endo@126.com

Hua Bian

Department of Endocrinology, Zhongshan Hospital, Fudan University, NO.180 Fenglin Road, Shanghai 200032, China.

Email: zhongshan_bh@126.com

Xinxia Chang

Department of Endocrinology, Zhongshan Hospital, Fudan University, NO.180 Fenglin Road, Shanghai 200032, China.

Email: changxinxiawei@163.com

## Supplementary Tables

Supplementary Table 1 The top enriched biological process (BP) terms enriched by DEPs.

| Term | Genes | p Value |
| --- | --- | --- |
| Cluster 1 |  |  |
| GO:0006958~complement activation, classical pathway | IGHG3, IGHG2, C9, MBL2 | 0.000274193 |
| GO:0010951~negative regulation of endopeptidase activity | SERPINA11, SERPINC1, SERPINE1 | 0.001501792 |
| GO:0042730~fibrinolysis | FGB, SERPINE1 | 0.019401993 |
| GO:0045087~innate immune response | FGB, IGHG3, IGHG2, MBL2 | 0.025384814 |
|  |  |  |
| Cluster 2 |  |  |
| GO:0006953~acute-phase response | CRP, SERPINA3, SERPINA1 | 0.000589528 |
| GO:0051546~keratinocyte migration | LRG1, KRT2 | 0.00555083 |
| GO:0030212~hyaluronan metabolic process | ITIH3, ITIH2 | 0.008315321 |
| GO:0030866~cortical actin cytoskeleton organization | LCP1, TLN1 | 0.028367594 |
|  |  |  |
| Cluster 3 |  |  |
| GO:0006508~proteolysis | FCN2, F12, PROZ, HABP2, CNDP1 | 0.00052878 |
| GO:0007155~cell adhesion | LGALS3BP, NCAN, IGFALS, HABP2, THBS4 | 0.001548686 |
| GO:0010897~negative regulation of triglyceride catabolic process | APOC3, GPLD1 | 0.004627717 |
| GO:1903028~positive regulation of opsonization | FCN2, CFP | 0.011072552 |
| GO:0006957~complement activation, alternative pathway | CFHR5, CFP | 0.015651854 |
|  |  |  |
| Cluster 4 |  |  |
| GO:0006956~complement activation | C3, FCN3, CFH, CFHR1, CFHR3 | 5.06168E-08 |
| GO:0030451~regulation of complement activation, alternative pathway | C3, CFH, CFI | 6.01239E-06 |
| GO:0006508~proteolysis | C3, FCN3, F9, CFH, CFI, MASP2, CTSD | 2.55595E-05 |
| GO:1903028~positive regulation of opsonization | C3, FCN3, MASP2 | 0.000131214 |
| GO:0006958~complement activation, classical pathway | C3, APCS, CFI, MASP2 | 0.00075888 |
| GO:0070527~platelet aggregation | PLEK, FN1, METAP1 | 0.002262133 |

Supplementary Table 2 Correlations between the other 86 proteins changed significantly throughout the entire postoperative follow-up and BMI, HOMA-IR, and hepatic fat content.

| DEPs | BMI | HOMA-IR | liver fat | HOMA-IR（adjusted by age, sex and BMI） | liver fat content (adjusted by age, sex and BMI） |
| --- | --- | --- | --- | --- | --- |
| NCAN |  |  |  |  |  |
| APOC4 |  | 0.507** | 0.408* | 0.511** | 0.531** |
| IGFBP3 |  |  |  |  |  |
| LCP1 |  |  |  |  |  |
| ALB | ‘-0.615*** |  |  |  |  |
| PPP1R9B |  | -0.421* |  | -0.496** |  |
| ALDOB |  | 0.624*** |  | 0.713*** |  |
| CFP |  |  |  |  |  |
| SERPINA1 | 0.422* |  |  | -0.56** |  |
| TLN1 |  |  |  |  |  |
| PROZ |  | 0.405* |  | 0.441* |  |
| KRT16 |  |  |  |  |  |
| SEC23B |  |  |  |  |  |
| JCHAIN | ’-0.392* |  |  |  |  |
| HABP2 |  |  |  |  |  |
| LPA |  |  |  |  |  |
| OAF |  |  |  |  |  |
| NDUFAF3 |  |  |  |  |  |
| APOC3 |  | 0.477** |  | 0.489* |  |
| CAMK2D |  |  |  |  |  |
| BTD |  |  | 0.668*** |  | 0.605** |
| C9 |  |  |  |  |  |
| IGFALS |  |  |  |  |  |
| SERPINA3 |  |  | -0.462* | -0.434* | -0.441* |
| ARPC1B |  |  |  |  |  |
| FHL1 |  |  |  |  |  |
| VCP |  |  | 0.428* |  |  |
| SERPINA11 | | -0.364* |  |  |  |
| DYNC1H1 | |  |  |  |  |
| CTSD |  |  | 0.615** |  | 0.643** |
| CNTN1 |  | -0.447* | -0.509** | -0.574** | -0.458* |
| CFHR5 | 0.436** |  |  |  |  |
| LGALSL |  |  |  |  |  |
| ICAM1 |  | 0.421* | 0.481* | 0.438* | 0.479* |
| MOB4 |  |  |  |  |  |
| PLEK |  |  | 0.399* |  |  |
| TNXA |  |  |  |  |  |
| METTL18 |  |  |  |  |  |
| METAP1 |  |  |  |  |  |
| MASP2 | 0.446** |  |  |  |  |
| COL6A3 |  |  |  |  |  |
| CRP |  |  |  |  |  |
| LIN7C |  |  |  |  |  |
| NPM1 |  |  | -0.587** |  | -0.581** |
| UQCRB |  | -0.396* |  |  |  |
| ICAM2 |  |  |  |  |  |
| CSN1S1 |  |  |  |  |  |
| ANG |  |  |  |  |  |
| GGH |  |  |  |  |  |
| GAS6 | 0.419* |  | 0.471* |  |  |
| ACP1 |  |  | -0.39* |  |  |
| F12 |  |  | 0.43* |  |  |
| YWHAG |  |  |  |  |  |
| MBL2 |  | -0.38* |  | -0.429* |  |
| CFHR1 | 0.414* |  |  |  |  |
| CFHR3 |  |  | 0.406* |  |  |
| CFH |  |  |  |  |  |
| C3 | 0.371* | 0.431* | 0.488** |  | 0.405* |
| CD2AP |  |  |  |  |  |
| SERPINE1 |  |  |  |  |  |
| THBS4 |  | 0.374* | 0.393* |  |  |
| EPS15 |  |  | 0.602** |  |  |
| PON3 | -0.54** |  | -0.441* |  |  |
| LAMP2 |  |  | 0.672*** |  | 0.478* |
| COLEC10 |  |  | -0.576** |  | -0.489* |
| F9 | 0.706*** |  |  |  |  |
| FGB | -0.34* |  | -0.394* |  |  |
| FCN2 |  |  | 0.488** |  |  |
| FUBP3 |  |  | -0.394* |  |  |
| LGALS3BP |  |  | 0.453* |  |  |
| CFI | 0.355* |  | 0.635*** |  | 0.448* |
| APCS | 0.453** | 0.415* | 0.559** |  | 0.51* |
| VTN |  | 0.43* | 0.726*** |  | 0.559** |
| SERPINC1 | -0.437** |  | -0.636*** |  | -0.507* |
| APOA4 | -0.412* |  |  |  |  |
| KRT2 | -0.475** |  |  |  |  |
| ACTR2 |  |  |  |  |  |
| GPLD1 |  |  |  |  | 0.532** |
| TMEM256 | -0.46** | -0.423* |  |  |  |
| ITIH2 |  | -0.376* |  | -0.41* |  |
| IGHV3-72 |  | -0.483** |  | -0.471* |  |
| IGHG3 |  | -0.444* |  | -0.553** |  |
| TPM1 |  | -0.439* |  |  |  |
| IGHG2 |  | -0.394* |  | -0.52** |  |
| LRG1 | 0.422* |  | -0.484* | -0.441* | -0.606** |
| FN1 |  |  | 0.584** |  | 0.408* |

Significant correlations with statistical significance are presented in the table.

*p<0.05, **p<0.01, ***p<0.001
